# Physiologic approach to diuresis in de-resuscitation phase in intensive care

**DOI:** 10.1186/s13054-020-02900-y

**Published:** 2020-05-28

**Authors:** Amos Lal, Juan Pablo Domecq Garces

**Affiliations:** Division of Pulmonary and Critical Care Medicine, Mayo Clinic, Rochester, MN USA

To the Editor,

We read with great interest the paper by Bissell et al. regarding impact of protocolized diuresis for de-resuscitation in the intensive care unit [1]. This document is timely in the era where intensivists are taking a more detailed focus on acute fluid resuscitation and de-resuscitation periods.

After reading the paper carefully, we would like to stress the fact that even when the protocol is showing interesting results, the protocol is still not based on physiologic data, something that is much needed at the time to care for critically ill patient. Indications for combination therapy were recommended only after reaching the maximum dose of furosemide or developing side effects (hypernatremia), when in fact, obese patients and patient on chronic diuretic therapy could benefit from adding thiazides as initial approach [2] due to high risk for distal tubular hypertrophy. Also, the electrolyte protocol does not comment on the importance of focusing on sodium and chloride both electrolytes independently and inversely associated with mortality in patients with volume overload. This is taking even more momentum now that hypertonic saline has been proposed as an effective intervention to increase diuretic efficiency [3].

Also we would like to point out a detail in the “Abstract” section; authors present post-shock fluid balance as median and interquartile range, whereas in the “Results” section (Table 3) the same outcomes were presented as average and standard deviation. This discrepancy affects the readability of results since there is a considerable difference between the two statistical measures. Also, upon cautious appraisal, it is difficult to interpret the results in the same table (Table 3 Clinical outcomes) when authors present post-shock fluid balance. For example, the 72-h fluid balance in the intervention cohort the average fluid balance is − 2257 mL with standard deviation being − 5676–920; does this mean that the standard deviation ranges from − 5676 to − 920 mL or − 5676 to + 920 mL? Although these may be visually minor appearing typographical inaccuracies, they greatly impact the understanding of the results from this paper.

**Table 3:** Clinical outcomes

Parameter	Historical cohort (*n* = 273)	Intervention cohort (*n* = 91)	*p* value
Clinical outcomes			
72 h fluid balance (mL)^a^	265 (− 2283–3025)	− 2257 (− 5676–920)	< 0.0001
48 h fluid balance (mL)^a^	309 (− 1267–2434)	− 1799(− 3884–1092)	< 0.0001
24 h fluid balance (mL)^a^	101 (− 963–1622)	− 692 (− 1833–697)	0.0002
Ventilator-free days (days)^a^	19 (10–22)	20 (15–23)	0.098
Overall adverse event^b,e^	74 (27.1)	37 (40.6)	0.015
Ventilator days (days)^a^	8 (5–13)	5 (5–12)	0.441
Furosemide to extubation (hours)^a^	70 (24–147)	58 (23–122)	0.282
Re-intubation rate^b^	57 (20.8)	17 (18.6)	0.652
ICU-free days (days)^a^	17 (7–21)	19 (13–22)	0.030
ICU days (days)^a^	8.6 (6.2–13.5)	8.1 (5.9–12.8)	0.513
In-hospital mortality^c^	44 (16.1)	5 (5.5)	0.008
Safety outcomes			
Bolus administration after furosemide^c^	4 (1.5)	0 (0)	0.576
Vasopressor administration after furosemide^b^	65 (23.8)	19 (20.9)	0.566
Tachyarrhythmia^b^	50 (18.3)	15 (16.4)	0.693
In-hospital mortality^c^	44 (16.1)	5 (5.5)	0.008
RRT receipt in ICU^c^	17 (6.2)	0 (0)	< 0.0001
RRT dependence at discharge^c^	14 (5.1)	0 (0)	0.025
Acute kidney injury^f^	62 (22.7)	22 (24.2)	0.775
Hypokalemia^c^	0	3 (3.3)	0.015
Hypernatremia^b^	19 (6.9)	19 (20.9)	0.001
Metabolic alkalosis^c^	3 (1.1)	1 (1.1)	1.000

^a^Wilcoxon rank sum, median (interquartile range)

^b^Chi-square test, number (percentage)

^c^Fisher’s exact, number (percentage)

^d^Student’s *t* test, average (standard deviation)

^e^Overall adverse event: serum creatinine rise, hypokalemia, hypernatremia, or metabolic alkalosis

^f^Acute kidney injury: serum creatinine 1.5 times baseline serum creatinine, serum creatinine increase of at least 0.3 mg/dL

We agree that protocolized diuresis is promising, and we would like to emphasize the need to maintain a physiologic approach for these protocols. As well, we would suggest incorporating into the original manuscript as an erratum will give a much clearer insight to the readers of the journal and greater scientific community.

## Authors’ response

Brittany D. Bissell; Javier A. Neyra

We thank Lal et al. for their comments [4] concerning our article on the impact of protocolized diuresis for de-resuscitation in the intensive care unit [1]. Regarding the typographical concerns expressed, all fluid balances reported in the “Results” section and specifically in Table 3 were evaluated via Wilcoxon rank sum and reported as median (interquartile range) (Clinical outcomes). The numbers and ranges are correct as reported. For the cited example in the intervention group, median 72 h fluid balance was − 2257 mL, with 25th percentile of − 5676 mL and 75th percentile of 920 mL.

We agree with the authors’ comments suggesting that physiologic data are important to consider for critically ill patients. We believe, however, there must be a balance between protocol complexity and personalization in therapeutics. This is best exemplified by the largest study modulating fluid balance in a broad critically ill population, the Comparison of Two Fluid-Management Strategies in Acute Lung Injury study [5]. Despite demonstrating significant improvement in mechanical ventilation duration and lung function, this protocol has had limited uptake by the critical care community to everyday practice. We hope to build upon our protocol to develop a more personalized approach to not only de-resuscitation, but volume management at large. To do so, however, two key areas must be researched: (1) loop diuretic pharmacodynamics in the critically ill with and without acute and/or chronic kidney impairment and (2) development of accurate, dynamic predictive models of diuretic responsiveness to simplify and identify target populations for protocolized approaches.

Lal et al. comments on combination diuretic therapy and electrolyte disturbances are pertinent, but it is prudent to understand that our protocol is intended for the broad intensive care unit population, not only those presenting with acute decompensated heart failure. Such patients represent a small portion of the critically ill population, noted by the 6.6–11.1% incidence of chronic loop diuretic usage and only 2.2–3.3% of admissions secondary to cardiac procedures in our study [1]. Predominate evidence for utilization of hypertonic saline (HTS) as a diuretic adjunct is restricted to patients presenting with heart failure, and current evidence would advise against such in the general critically ill population given that chloride is directly, rather than inversely, associated with mortality while the correlation with sodium may be biphasic [6, 7]. Until further data are available, HTS remains unlikely a feasible addition to broad protocolized approaches at this time. We agree that in patients with chronic exposure to loop diuretics, initial or early combination therapy including thiazides should be considered, particularly given the incidence of hypernatremia in our study.

## Data Availability

Not applicable
